# Fluctuation Relations for Dissipative Systems in Constant External Magnetic Field: Theory and Molecular Dynamics Simulations

**DOI:** 10.3390/e23020146

**Published:** 2021-01-25

**Authors:** Alessandro Coretti, Lamberto Rondoni, Sara Bonella

**Affiliations:** 1 Department of Mathematical Sciences, Politecnico di Torino, Corso Duca degli Abruzzi 24, I-10129 Torino, Italy; alessandro.coretti@polito.it (A.C.); lamberto.rondoni@polito.it (L.R.); 2 Centre Européen de Calcul Atomique et Moléculaire (CECAM), École Polytechnique Fédérale de Lausanne, Batochime, Avenue Forel 2, 1015 Lausanne, Switzerland; 3 Istituto Nazionale di Fisica Nucleare, Sezione di Torino, Via P. Giura 1, I-10125 Torino, Italy

**Keywords:** statistical mechanics, time reversibility, magnetic field, Fluctuation Relations, molecular dynamics

## Abstract

We illustrate how, contrary to common belief, transient Fluctuation Relations (FRs) for systems in constant external magnetic field hold without the inversion of the field. Building on previous work providing generalized time-reversal symmetries for systems in parallel external magnetic and electric fields, we observe that the standard proof of these important nonequilibrium properties can be fully reinstated in the presence of net dissipation. This generalizes recent results for the FRs in orthogonal fields—an interesting but less commonly investigated geometry—and enables direct comparison with existing literature. We also present for the first time a numerical demonstration of the validity of the transient FRs with nonzero magnetic field via nonequilibrium molecular dynamics simulations of a realistic model of liquid NaCl.

## 1. Introduction

It is well known that the evolution equations of charged systems subject to an external magnetic field (in this work, we focus on classical systems) are not invariant under the standard time-reversal transformation, $M_{s}$, defined via inversion of the momenta

(1) $$M_{s}\left(r,p\right)=\left(r,-p\right)\;,\;\forall \left(r,p\right)≐\Gamma \in M$$

combined with the change t→−t. Here *t* is the time variable and, in the equation above, Γ is a point in the phase space M of an *N*-particle system, with positions $r={\left\{r_{i}\right\}}_{i=1}^{N}$ and momenta $p={\left\{p_{i}\right\}}_{i=1}^{N}$. This fact originated the idea that these systems require special treatment when discussing properties based on time reversibility. In particular, because the currents (and therefore the magnetic field that they generate) are reversed under $M_{s}$, classical text books [1,2] as well as current literature [3] report statistical relationships in the presence of a magnetic field using pairs of systems with identical interparticle interactions but under magnetic fields of opposite signs. For example, the Onsager reciprocal relations were adapted by Casimir to relate cross-transport coefficients of systems under opposite magnetic fields [4]. Similarly, Kubo [5] derived symmetry properties of time-correlation functions of two such systems. In nonequilibrium statistical mechanics, results for currents, response, and Fluctuation Relations (FRs) are typically presented under the same conditions [3,6,7,8]. All these results are to be contrasted with their “standard” counterparts, i.e., those derived in the absence of magnetic fields, that refer to a single system.

The situation described above is somewhat unsatisfactory for two main reasons. The first is conceptual: the introduction of a second system, while physically correct, blurs the distinction between the system and its external environment. Indeed, in the evolution equations of the system, the magnetic field typically appears as an external agent whose physical origin (e.g., moving charges originating a current) is not associated to active degrees of freedom in the dynamical system. Its inversion then implicitly implies extending the system to include the sources of the magnetic field, applying $M_{s}$ to the extended system, and then forgetting again about the additional degrees of freedom. The second reason is practical: this commonly adopted approach reduces the predictive power of the statistical relationships mentioned above. For example, within linear response theory, null values of transport coefficients in experiments concerning a single system in a given magnetic field cannot be predicted based on symmetry properties of the time-correlation functions. Similarly, in the context of nonlinear response, which includes the Fluctuation Relations [9,10,11,12,13], null cumulants of the dissipation cannot be identified via symmetry [3].

Recently however, it was demonstrated that, for systems in a constant external magnetic field, these difficulties can be overcome, recovering the full predictive power of statistical mechanics. The starting observation for these recent developments is that invariance of the Hamiltonian (and hence of the dynamical system) under Equation (1) is a sufficient but not necessary condition for establishing the properties mentioned above. Following a known approach in nonequilibrium statistical mechanics, alternative time-reversal operators—that do not necessitate inversion of the magnetic field—can be introduced [14,15,16,17,18] and used instead of $M_{s}$ to reinstate standard proofs. These new symmetries lack the intuitive property of retracing the coordinates in the backward propagation in pairs of trajectories with opposite momenta, but they nonetheless identify pairs of trajectories with opposite values of relevant observables (e.g., elements of the diffusion tensor or of the instantaneous dissipation) and their physical effects can be predicted and measured. This was illustrated numerically for the case of time-correlation functions in Refs. [16,17] and for Fluctuation Relations in the presence of orthogonal electric and magnetic fields in Ref. [19]. The lack of experimental evidence of the violation of the Onsager reciprocal relations [20] might also be explained via these generalized time-reversal operators.

In this paper, we continue the investigation of the nonequilibrium behavior of classical charged systems in external magnetic and electric fields and generalize and consolidate previous work. We reconsider, in particular, FRs and extend the results presented in Ref. [19] in two important ways. First, in contrast to the model considered in that paper, which had no net dissipation because the electric and magnetic field were orthogonal, here we consider the case of parallel fields. This leads to net currents and the presence of the corresponding dissipation, as standard in the investigation of FRs [11,13]. Our results can then be contrasted directly with existing work [3]. To prove the validity of FRs also with this set up we (trivially) extend to the case of thermostatted evolution two generalized time-reversal operators, different from the ones used in Ref. [19], and introduced in Ref. [17]. The use of these operators makes it possible to demonstrate the validity of a transient FR that benefits from a fully single magnetic field description and therefore takes us one step further in the single system approach to the statistical properties of objects in constant magnetic field. The second addition to our previous work is the corroboration of the transient FRs by molecular dynamics simulations of a realistic model of NaCl. While these numerical results are not strictly surprising given the mathematical proof of the FRs, they are nonetheless nontrivial. The onset and the observation of FRs in realistic models of condensed-phase systems and within the size and time scales accessible in the simulation requires an accurate integration of the evolving equations that fully enforces the formal properties (e.g., generalized time-reversibility) required by the proof. This is made possible in this work by the use of a recently developed symplectic and time-reversible algorithm that includes a modified Nosé–Hoover thermostat to enforce constant temperature [21]. Furthermore, since FRs require to consider values of the dissipation on the tails of the probability distribution, ensuring a satisfactory signal-to-noise ratio can be problematic. The combination of accurate integration of the system and sufficient statistics, however, made it possible to directly show the odd parity of the dissipation under the proposed generalized time-reversal operators and verify the validity of the transient FR for a representative value of the electric field.

The paper is organized as follows. In Section 2, we start by setting up our dynamical system and discussing its properties. We then introduce the relevant generalized time-reversal symmetries and compute the dissipation function and the transient FRs. In the main text, we recall only the key definitions and properties necessary to proceed. A detailed set of definitions, together with a summary of the derivation of the FRs can be found in Appendix A, while the explicit calculation of the instantaneous dissipation is provided in Appendix B. Section 3 provides details on the NaCl simulation and illustrates the theoretical statements via appropriate numerical results.

## 2. Theory

Let us consider *N* particles of charge $q_{i}$ and mass $m_{i}$ in three dimensions and in the presence of external uniform and static electric and magnetic fields. The Hamiltonian of the system is

(2) $$\begin{array}{cc} H(\Gamma ) & =H_{0}\left(\Gamma \right)-\sum_{i=1}^{N}q_{i}E\cdot r_{i}=\\ \; & =\sum_{i=1}^{N}\frac{{\left(p_{i}-q_{i}A\left(r_{i}\right)\right)}^{2}}{2m_{i}}+\sum_{i,j<i}^{N}V\left(r_{ij}\right)-\sum_{i=1}^{N}q_{i}E\cdot r_{i} \end{array}$$

In the equation above, A(r) is the vector potential associated to the magnetic field $B=\nabla_{r}\times A\left(r\right)$, E is the electric field, and $V(r_{ij})$ a pairwise additive interaction potential, depending only on the modulus of the distance between particles: $r_{ij}=\left|r_{i}-r_{j}\right|$. We set $E=(0,0,E_{z})$ and $B=(0,0,B_{z})$, i.e., the fields are parallel and oriented along the *z*-axis. In the Coulomb gauge ($\nabla_{r}\cdot A\left(r\right)=0$), a valid choice for the vector potential is $A\left(r\right)=B_{z}/2\left(-y,x,0\right)$. The choice of the gauge does not affect the discussion below since it cannot affect the evolution equations, see also [18]. This setting, while not completely general, is typically adopted to discuss the time-reversal properties of systems in external magnetic fields [3,6,22,23] and it describes relevant physical situations. In particular, in contrast with previous work [19], this orientation of the electric and magnetic fields ensures the presence of dissipation in the system and this is the framework in which FRs and their corollaries are generally considered.

The Hamiltonian in (2) generates the motion of the system. The notation indicates that, in the following, we shall consider as the equilibrium the state of the system when it is subject to the internal interactions and the external magnetic field. The electric field then acts as the external perturbation driving the system out of equilibrium. This definition of the equilibrium state (slightly unusual in that it includes an external field) is viable because the magnetic field does not perform work on the system. To prevent uncontrolled heating up of the system when the external electric field is active, we introduce a Nosé–Hoover thermostat. In particular, we shall consider a modified version of the thermostat that was introduced to account for the presence of magnetic and electric fields in Ref. [21]. The corresponding dynamical system is

(3) $$\begin{array}{cc} \frac{dx_{i}}{dt} & =\frac{p_{i}^{x}}{m_{i}}+\omega_{i}y_{i}\\ \frac{dy_{i}}{dt} & =\frac{p_{i}^{y}}{m_{i}}-\omega_{i}x_{i}\\ \frac{dz_{i}}{dt} & =\frac{p_{i}^{z}}{m_{i}}\\ \frac{d\ln s}{dt} & =\xi \end{array}\;\;\begin{array}{cc} \frac{dp_{i}^{x}}{dt} & =F_{i}^{x}+\omega_{i}\left(p_{i}^{y}-m_{i}\omega_{i}x_{i}\right)-\xi \left(p_{i}^{x}+m_{i}\omega_{i}y_{i}\right)\\ \frac{dp_{i}^{y}}{dt} & =F_{i}^{y}-\omega_{i}\left(p_{i}^{x}+m_{i}\omega_{i}y_{i}\right)-\xi \left(p_{i}^{y}-m_{i}\omega_{i}x_{i}\right)\\ \frac{dp_{i}^{z}}{dt} & =F_{i}^{z}+q_{i}E_{z}-\xi p_{i}^{z}\\ \frac{d\xi }{dt} & =\frac{1}{\tau_{\mathrm{NH}}^{2}}\left[\frac{K\left(\Gamma \right)-K^{*}}{K^{*}}\right]≐\frac{\delta K(\Gamma )}{\tau_{\mathrm{NH}}^{2}} \end{array}$$

where $\omega_{i}=q_{i}B_{z}/2m_{i}$ is the cyclotron frequency of particle *i* and $\tau_{\mathrm{NH}}$ is the characteristic time of the thermostat. In the system above, the evolution of the Nosé variable ξ is governed by the difference between the target kinetic energy $K^{*}=Gk_{B}T/2$ and the instantaneous microscopic estimator of the kinetic energy $K\left(\Gamma \right)=\sum_{i,\alpha }\frac{{\left(p_{i}^{\alpha }-q_{i}A^{\alpha }\left(r_{i}\right)\right)}^{2}}{2m_{i}}$, with α=x,y,z. (In the definition of the target kinetic energy, $k_{B}$ is the Boltzmann constant, *T* the target temperature, and *G* the number of degrees of freedom of the system.) The dynamical system above admits a conserved quantity analogous to the Nosé–Hoover constant of the motion and given by $H_{\mathrm{NH}}\left(\Gamma ,\xi ,s\right)=H\left(\Gamma \right)+K^{*}\left[\tau_{\mathrm{NH}}^{2}\xi^{2}+2\ln s\right]$. The characteristics of Equation (3) can be summarized as follows. Firstly, note that when $B_{z}=0$ (i.e., $\omega_{i}=0$ for all particles) and $E_{z}=0$, the dynamical system reduces to a standard Nosé–Hoover thermostatted system. In this case, the Hamiltonian momenta are trivially proportional to the particle’s velocities, the kinetic energy estimator of the temperature reduces to the usual prescription, and the terms proportional to the Nosé variable ξ in the time derivatives of the momenta are also standard. On the other hand, when $B_{z}\neq 0$ (but $E_{z}=0$), the relationship between the particle’s velocities and momenta includes a contribution arising from the magnetic field, as indicated in the first three equations of (3). Furthermore, the estimator of the temperature (a well defined quantity because the magnetic field does not perform work) is adapted via the use of the instantaneous kinetic energy K(Γ), which employs the velocities. This is a natural choice for a system in external magnetic because the Lorentz force involves velocities, not momenta. Moreover, as shown for the reader’s convenience in Appendix B, even in the presence of a nonzero vector potential, the canonical average value of kinetic energy is proportional to the temperature. Adopting the instantaneous kinetic energy as the regulator of the temperature, the dynamics of ξ is also governed by changes in the velocities (not the momenta) of the particles. This is reflected in the evolution of the particle’s momenta in (3), where the term proportional to the Nosé variables is easily recognizable as the product between the mass and the velocity. As proved in Ref. [21], when no electric field is present, the dynamics samples the equilibrium distribution

(4) $$f_{0}\left(\Gamma ,\xi \right)=Z^{-1}\exp\left[-\beta H_{0}\left(\Gamma \right)\right]\exp\left[-\frac{G\tau_{\mathrm{NH}}^{2}\xi^{2}}{2}\right]$$

where Z is the partition function and $\beta ={\left(k_{B}T\right)}^{-1}$. As in standard Nosé–Hoover dynamics, the variable *s* does not enter directly in the dynamics of the physical variables and in the distribution. Furthermore, again as in the standard case, the marginal probability obtained integrating Equation (4) with respect to ξ is the canonical density, in magnetic field, for the physical variables. Finally, when both the magnetic and electric fields are active, Equation (3) enables to maintain the instantaneous kinetic energy (a well defined quantity both at equilibrium and far from it) close to a predefined reference value that acts as a proxy for the temperature, a more problematic concept out of equilibrium [24,25]. Note that the systematic drift velocity induced by the electric field is not subtracted from instantaneous kinetic energy. For the simulation set up chosen in the following (see Section 3) the component of the velocity of the center of mass in the direction of the electric field is constantly null and for more general systems it is usually small.

Let us now discuss the behavior under time reversal of the dynamical system in Equation (3). Direct inspection shows that, as expected, standard time reversal does not hold even considering a natural extension which includes the Nosé–Hoover auxiliary variables by leaving *s* unchanged and changing the sign of ξ. This transformation will be indicated in the following as

(5) $$M_{s}^{\mathrm{ext}}\left(\Gamma ,s,\xi \right)=\left(x,y,z,-p^{x},-p^{y},-p^{z},s,-\xi \right)$$

The violation of $M_{s}^{\mathrm{ext}}$ is due to the coupling between coordinates and momenta induced by the magnetic field and seems to imply that a standard treatment of equilibrium and nonequilibrium statistical mechanics relationships based on time reversal is indeed impossible. However, the proof of these relationships requires the existence of (at least) one valid time-reversal operator and the even parity of the equilibrium distribution under this operator, but it does not prescribe the specific form of the operator and, in particular, it does not fix it to $M_{s}$ or $M_{s}^{\mathrm{ext}}$. In fact, generalized time-reversal operators, different from $M_{s}$, have already been used in the literature to investigate the equilibrium and nonequilibrium statistical mechanics of deterministic particle systems [26]. This approach has recently been extended to constant-energy systems in external magnetic and electric fields via the introduction of a set of time-reversal symmetries valid in different conditions (e.g., different orientation of the fields) [16,17]. In Ref. [19] these symmetries where adapted—for orthogonal orientation of the fields—to the isokinetic and Nosé–Hoover dynamics. Based on these works, new generalized time-reversal symmetries can be defined also for the case, considered in this paper, of Nosé–Hoover evolution in parallel (uniform and time independent) electric and magnetic fields. In particular, let us denote as $M_{\mathrm{ext}}$ the extended phase space spanned by the dynamical system (3), and as $U_{t}$ the associated time-evolution operator for a time *t*. Generalized time-reversal operators in this extended phase space are defined, in complete analogy with what is done in the physical phase space, as involutions $M_{\mathrm{ext}}$ satisfying

(6) $$U_{-t}X=M_{\mathrm{ext}}U_{t}M_{\mathrm{ext}}X\;\forall t\in R\;,\;\forall X\in M_{\mathrm{ext}}$$

where X=(Γ,s,ξ) is a point of the extended phase space. Two operators that satisfy Equation (6) can be defined for (3):

(7a) $$\begin{array}{cc} M_{\mathrm{ext}}^{\left(3\right)}\left(\Gamma ,s,\xi \right) & =(-x,y,z,p^{x},-p^{y},-p^{z},s,-\xi ) \end{array}$$

(7b) $$\begin{array}{cc} M_{\mathrm{ext}}^{\left(4\right)}\left(\Gamma ,s,\xi \right) & =(x,-y,z,-p^{x},p^{y},-p^{z},s,-\xi ) \end{array}$$

Invariance of the dynamical system under the transformations above and time inversion can be verified easily by direct inspection of Equation (3). The notation adopted in Equation (7) reflects the nomenclature introduced in Ref. [17] where related symmetries—established in the absence of a thermostat—were first introduced. Note that the equilibrium density Equation (4) is even under these transformations. As mentioned above, the validity of the time-reversal operators defined in Equation (7) and the even parity of the equilibrium probability density of the system is a sufficient condition to reinstate standard proofs of relevant statistical mechanics relationships. Notably, these new time-reversal symmetries act only on the active degrees of freedom of the dynamical system and do not require inversion of the magnetic field. Based on these symmetries, we can then derive interesting results within a single-system (single magnetic field) discussion of the dynamics. For example, following the derivation in Ref. [17], it can be shown within linear response theory that the yz and xz components of the diffusion and conductivity tensors of the system must be zero.

In the following, we shall consider the implications of the newly introduced time-reversal operators on nonequilibrium properties of the system, focusing in particular on the transient fluctuation relation. These relations have been discussed in a variety of publications, reviewed for instance in [11,13,27]. In the following, we shall introduce and discuss only the concepts and quantities more directly related to our calculations. A review of relevant notation and some mathematical proofs are also available in the Appendices. The key quantity in the transient fluctuation relation is the instantaneous dissipation function that, in the extended phase space, is defined as

(8) $$\Omega^{\left(0\right)}\left(X\right)≐-\dot{X}\cdot \nabla_{X}\ln f_{0}-\Lambda \left(X\right)$$

where $\Lambda \left(X\right)=\nabla_{X}\cdot \dot{X}$ is the phase-space expansion rate and $f_{0}$ has been defined in Equation (4). Substitution of Equation (4) in the definition above shows, after some algebra reported in Appendix B and similar to the developments in Ref. [19], that $\dot{X}\cdot \nabla_{X}\ln f_{0}=\beta \xi 2K\left(\Gamma \right)-\beta \sum_{i=1}^{N}q_{i}{\dot{r}}_{i}\cdot E-G\xi \delta K\left(\Gamma \right)$, with δK(Γ) defined as in the last equation of Equation (3). Furthermore, Λ(X)=−Gξ. Combining these results in Equation (8) we obtain

(9) $$\Omega^{\left(0\right)}\left(X\right)=V\beta J\left(\Gamma \right)\cdot E$$

where V is the volume of the system and $J\left(\Gamma \right)=V^{-1}\sum_{i=1}^{N}q_{i}{\dot{r}}_{i}$ is the microscopic current. The average dissipation over a finite time-leg τ is defined as

(10) $${\bar{\Omega^{\left(0\right)}}}_{0,\tau }\left(X\right)≐\frac{1}{\tau }\int_{0}^{\tau }ds\Omega^{\left(0\right)}\left(U_{s}X\right)$$

In the definition above, $\Omega^{\left(0\right)}\left(U_{s}X\right)$ indicates that the observable is averaged along a trajectory of duration *s* starting from the initial conditions *X*, and the notation ${\bar{\Omega^{\left(0\right)}}}_{0,\tau }\left(X\right)$ on the left hand side underlines that—due to the finite time over which the average is taken—the result of the integral depends on the initial conditions.

**Figure 1:**
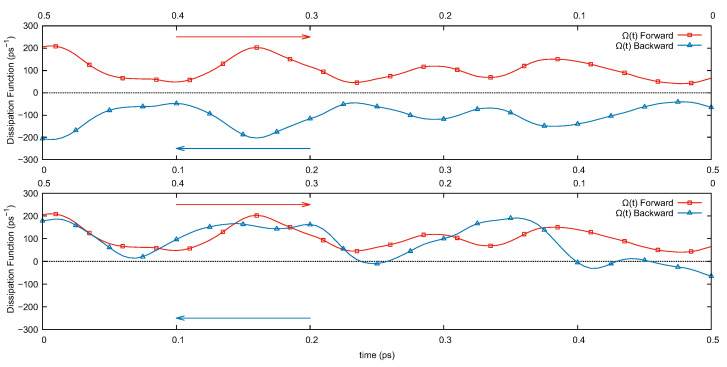
Instantaneous dissipation function from Equation (8) for 500fs of the “forward” evolution (red curve, open squares) and for 500fs of the “backward” trajectory (blue curve, open triangles) obtained via $M_{\mathrm{ext}}^{\left(3\right)}$ (upper panel). The opposite values of the dissipation demonstrate the odd signature under the generalized time-reversal transformation used. The same behavior is not observed when the backward trajectory is obtained applying $M_{s}^{\mathrm{ext}}$ (bottom panel) as the presence of the magnetic field breaks the symmetry of the system under this transformation. Results are for the nonequilibrium simulation set-up described in Section 3 for liquid NaCl.

As required for the proof of the transient FRs and expected from the equations, in this system the instantaneous dissipation is odd under Equation (7). In Figure 1 (top panel), we show the behavior of this quantity under $M_{\mathrm{ext}}^{\left(3\right)}$ (the behavior under $M_{\mathrm{ext}}^{\left(4\right)}$ is the same). In the figure, $\Omega^{\left(0\right)}\left(t\right)$ is computed along a “forward” trajectory (in red), and along the “backward” trajectory (in blue) identified by $M_{\mathrm{ext}}^{\left(3\right)}$ for a molecular dynamics run of liquid NaCl with realistic interactions (the details of the simulation are provided in the next section). The two curves are obtained as follows: the dynamical system (3) is evolved for 500fs (“forward” trajectory) and $\Omega^{\left(0\right)}\left(t\right)$ is computed along the trajectory. The operator $M_{\mathrm{ext}}^{\left(3\right)}$ is then applied to the phase-space point obtained at the end of the evolution and the system is evolved again via Equation (3) for 500fs starting from the transformed point (“backward” trajectory). Along this trajectory, we compute again $\Omega^{\left(0\right)}\left(t\right)$. The odd parity of the dissipation is apparent from the figure. As a curiosity, in the bottom panel of Figure 1, we show the results for calculations in which the “backward” trajectory corresponds to standard time-reversal in the extended phase space. The figure clearly shows the lack of a specific signature for the dissipation under this symmetry, as expected from the theory. The different behavior of the dissipation under the two symmetries is clearly apparent over the relatively short propagation times reported in the figure. For larger systems—where chaotic motion dominates—and longer times, verifying the validity of an expected time-reversal symmetry might be problematic as numerical noise corrupts the symmetry of the forward and backward signals. The stability and time-reversal properties of the integration algorithm play a role on the propagation times for which symmetries can be verified and this is one of the reasons why, as mentioned in the next section, we have employed the symplectic algorithm introduced in Ref. [21] in our calculation. The effects of numerical precision on the propagation might also be tested, to some extent, by changing the time step and verifying the stability of the results obtained for the trajectories or the dissipation function under the action of the different time-reversal operators.

Having computed and characterized the dissipation function, we now move to the associated transient fluctuation relation. Details of the definitions and derivation of the FR can be found in Appendix A. This relation provides an explicit expression for the ratio of the initial probabilities to find the average dissipation function, ${\bar{\Omega^{\left(0\right)}}}_{0,\tau }$ in a neighborhood of size δ of the values *A* and of −A. Defining the subset of the phase space where the average dissipation takes values in the interval ${\left(\pm A\right)}_{\delta }=\left(\pm A-\delta ,\pm A+\delta \right)$ as ${\left\{{\bar{\Omega^{\left(0\right)}}}_{0,\tau }\right\}}_{{\left(\pm A\right)}_{\delta }}$, the transient FRs are given by [13,28]

(11) $$\begin{array}{c} \frac{\mu_{0}\left({\left\{{\bar{\Omega^{\left(0\right)}}}_{0,\tau }\right\}}_{{\left(-A\right)}_{\delta }}\right)}{\mu_{0}\left({\left\{{\bar{\Omega^{\left(0\right)}}}_{0,\tau }\right\}}_{{\left(+A\right)}_{\delta }}\right)}=\frac{\int_{{\left\{{\bar{\Omega^{\left(0\right)}}}_{0,\tau }\right\}}_{{\left(-A\right)}_{\delta }}}f_{0}\left(X\right)dX}{\int_{{\left\{{\bar{\Omega^{\left(0\right)}}}_{0,\tau }\right\}}_{{\left(+A\right)}_{\delta }}}f_{0}\left(X\right)dX}=\exp\left[-\tau [A+\epsilon (\delta ,A,\tau )]\right] \end{array}$$

where ϵ is a correction term obeying |ϵ(δ,A,τ)|≤δ. Previous discussions of (transient albeit long-time limit) FRs in the presence of aligned static external electric and magnetic fields [29], relied on the classical time-reversal and employed averages with respect to equilibrium distributions associated to opposite magnetic fields. The existence of $M_{\mathrm{ext}}^{\left(3\right)}$ and $M_{\mathrm{ext}}^{\left(4\right)}$, however, enables to repeat the proof of the relation in a single-system picture. The proof, detailed in Appendix A, follows the same steps as in the standard derivation, but invokes the new operators instead of $M_{s}$ where appropriate. In the next section, the validity of this single-system relation is illustrated via molecular dynamics simulations.

## 3. Simulations and Results

In the following, the theoretical results presented in the previous section are illustrated and further validated via molecular dynamics simulations of a realistic model of liquid NaCl. The simulated system consists of 125 Na${}^{+}$ and 125 Cl${}^{-}$ ions in a cubic box of side 20.9Å. This corresponds to a physical density $\rho =1.3793\;g\;{\mathrm{cm}}^{-3}$ (or ionic number density of 0.0275Å${}^{-3}$). The temperature is set to T=1550K. Pair interactions are modeled using a generalized Huggins–Mayer potential, with the parameters proposed by Tosi and Fumi in Ref. [30] and ionic charges $q_{\mathrm{Na}}=+1\;e$ and $q_{\mathrm{Cl}}=-1\;e$ (with *e* elementary charge) for sodium and chloride, respectively. The magnetic field, directed along the *z*-axis, is set to to the value of B=(0,0,50)cu (cu stands for code units: a detailed description of these units and of the conversion factors used in the code can be found in Ref. [21]), corresponding to approximately $B_{z}=5\times 10^{6}\;T$. The intensity of the field—huge on experimental scales—is not unusual in the context of molecular dynamics simulations of interacting systems in external fields [21,31,32,33,34] and is dictated by the relative strength of the external to the interparticle forces. In particular, to observe appreciable effects of the external field in a reasonable simulation time, the ratio between the average interparticle forces and the average Lorentz forces has to be around one. The chosen intensity of the magnetic field results in a value of this ratio approximately equal to 0.2. Note that the magnetic field is part of the equilibrium Hamiltonian for our system. In the driven simulations, the electric field—also directed along the *z*-axis—is chosen to be E=(0,0,10)cu, corresponding approximately to $E_{z}=1\times 10^{9}\;V\;m^{-1}$. With this choice of the field, the ratio between the average Lorentz forces and the average electrical drift forces (absolute value) is circa 1.

In the simulations, periodic boundary conditions are enforced in all directions. The evolution Equation (3) are integrated via a straightforward adaptation to the case of parallel (static and constant) magnetic and electric fields of the symplectic algorithm proposed in Ref. [21] for the evolution of a thermalized classical charged system in perpendicular fields. The long-range Coulombic interactions are treated using the Ewald summation method with an Ewald smearing parameter α=0.1 in code units. A timestep of δt=0.25fs is chosen for all the simulations, ensuring that the fluctuations of the Nosé conserved quantity are essentially zero. The characteristic time of the generalized Nosé–Hoover thermostat is set to 5fs, in accordance with the prescriptions for the value of $\tau_{\mathrm{NH}}$ given in Ref. [35].

**Figure 2:**
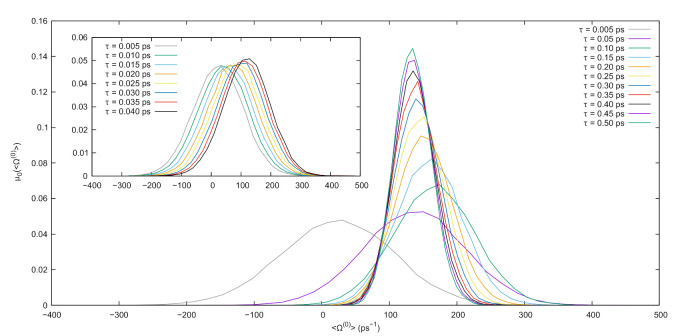
Probability distribution functions estimated from the normalized histogram of the average dissipation function computed in nonequilibrium runs starting from $5\times 10^{4}$ decorrelated equilibrium configurations for different values of τ. The main plot shows τ ranging from 0.005 to 0.5 ps, while the inset shows the trend for the low values of τ ranging from 0.005 to 0.04 ps, i.e., just after the switching on of the electric field that acts as the dissipative, nonequilibrium force.

The results discussed in this work are obtained via the following simulation scheme. Initial conditions are fixed by placing the ions in a BCC lattice, and sampling initial velocities from the Maxwell–Boltzmann distribution corresponding to the target temperature. A preliminary equilibration run of 25ps is then executed at null electric field to enforce the target temperature via the generalized Nosé–Hoover thermostat. Following this, a long equilibrium simulation (E=0) is performed to sample the equilibrium probability distribution $f_{0}$. In this run, phase-space configurations are sampled every 500fs (a sufficient interval to ensure decorrelation) along a trajectory of total length equal to 25ns, yielding a sample of $5\times 10^{4}$ decorrelated configurations. From each of these configurations, a nonequilibrium run is started where the electric field is switched on to the reference value of E=(0,0,10)cu. The average dissipation function, Equation (10), is computed along each driven trajectory for a set of values of τ, ranging from 5 to 500fs. Probability distribution functions (PDFs) for the possible values of the average dissipation at different times are then extracted through a histogramming process. Results for the PDFs are presented in Figure 2, showing the typical shifting and narrowing around the driven value of the dissipation as the simulation time lengthens.

**Figure 3:**
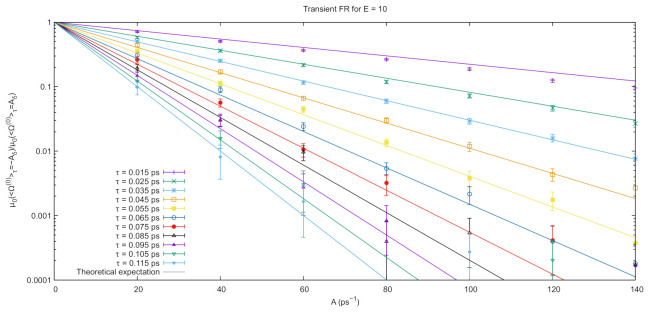
Simulation results for the transient fluctuation relation from the simulations described in Section 3. The points represent the ratio between probabilities (on the *y*-axis) of obtaining opposite values of the average dissipation (*A*, on the *x*-axis) at different values of τ from Figure 2. The statistical error is obtained dividing the $5\times 10^{4}$ nonequilibrium runs in 50 blocks of $10^{3}$ runs each and computing the normalized histogram for each of them. The error reported on the plot is obtained from the standard deviation on the single bin of the histograms relative to different blocks. Points are the simulation results, while the solid lines represent theoretical expectations. Parameters obtained from exponential fits performed on this set of numerical results are reported in Table 1. Note the logarithmic scale on the *y*-axis.

From the probabilities of opposite values of the average dissipation functions, it is possible to check Equation (11) for the system under investigation. Results are reported in Figure 3 for τ ranging from 0.015ps to 0.115ps together with the corresponding theoretical expectations, computed from Equation (11). As expected, the agreement between the theoretical result (solid curves) and the molecular dynamics calculation suffers as the length of the simulation and the value of *A* increase. The exponential behavior of the calculated quantities is, however, apparent and the agreement between the two sets of data is very good. To further quantify this agreement, in Table 1, we show the values for τ obtained from exponential fits performed on the numerical results and compare them with the exact value. In this case too, the agreement is very good within error bars.

## 4. Concluding Remarks

In this work, we have demonstrated that, and illustrated how, the transient FR can be actually verified in nonequilibrium molecular dynamics simulations of particles subject to magnetic and electric fields, and which are Nosé–Hoover thermostatted. The dissipation function, as well as the deterministic thermostat, have been expressed for the case in which electric and magnetic fields are parallel to each other. Although the applicability of generalized time-reversal symmetries implicitly implies the validity of the (transient) FR, based on the Nosé–Hoover canonical initial equilibrium distribution, the actual possibility of verifying it in a concrete, realistic simulation is not obvious. In the first place, to the best of our knowledge, this test has never been performed in presence of a magnetic field, which substantially modifies the dynamics of particles. In the second place, a verification of the FR may be hindered by the combination of scarce statistics and noise in the signal. Indeed, while the thermal noise becomes irrelevant at long observation (averaging) times, such long times drastically reduce the statistics of negative dissipations. Our simulations prove that the delicate balance allowing the verification of the FR, can be achieved for systems of moderately large size. Future developments will address the steady-state FR, which requires further conditions to be verified besides the time symmetry of the dynamics and of the initial phase-space probability distribution [13,28,36,37].

## Figures and Tables

**Table 1:** Comparison between the expected value of τ and the one obtained from the exponential fits performed on the numerical results in Figure 3.

$\tau_{\exp}$ (ps)	$\tau_{\mathrm{simul}}$ (ps)
0.015	0.016±0.001
0.025	0.026±0.001
0.035	0.035±0.001
0.045	0.044±0.001
0.055	0.054±0.001
0.065	0.063±0.002
0.075	0.073±0.002
0.085	0.086±0.003
0.095	0.092±0.003
0.105	0.103±0.003
0.115	0.116±0.005

## Data Availability

The used and analyzed datasets during the present study are available from the corresponding author on reasonable request.

## Abbreviations

The following abbreviations are used in this manuscript:

MDPI	Multidisciplinary Digital Publishing Institute
DOAJ	Directory of open access journals
FR	Fluctuation Relation
MD	Molecular Dynamics
PDF	Probability Distribution Function

## Appendix A. Mathematical Proof of the Transient Fluctuation Relation

In what follows, we summarize the derivation of the transient FRs for general systems, stressing the role of time-reversal symmetry in the proof. General discussions about the FRs can be found, for example, in Refs. [27,28,38,39,40].

Let us consider a dynamical system

(A1) $$\dot{X}=G\left(X\right)$$

where X∈M is a point in the generic phase space M and G:M→M is a vector field. For any observable Ψ∈O—where O is the space of all the classical observables—and time interval [t,t+τ] with τ>0, we define

(A2) $$\Psi_{t,t+\tau }\left(X\right)≐\int_{t}^{t+\tau }ds\Psi \left(U_{s}X\right)$$

which is also an observable. In the equation above, $U_{s}$ is the propagator for a time *s* associated to *G*. The time average over a time τ of Ψ is given by ${\bar{\Psi }}_{t,t+\tau }\left(X\right)≐\tau^{-1}\Psi_{t,t+\tau }\left(X\right)$. For any interval (a,b)⊂R we denote by ${\left\{\Psi \right\}}_{\left(a,b\right)}$ the set of phase-space points such that Ψ takes values in (a,b):

(A3) $$M⊃{\left\{\Psi \right\}}_{\left(a,b\right)}≐\left\{X\in M:\Psi \left(X\right)\in \left(a,b\right)\right\}$$

Let M be endowed with a probability measure $\mu_{0}$ of density $f_{0}$, at time t=0, so that $d\mu_{0}\left(X\right)=f_{0}\left(X\right)dX$ is the probability of an infinitesimal volume element around *X*. In what follows $f_{0}$ is not (necessarily) stationary under $U_{t}$. The probability of finding the value of Ψ in a given interval (a,b) at time t=0 is given by

$$\mu_{0}\left({\left\{\Psi \right\}}_{\left(a,b\right)}\right)=\int_{{\left\{\Psi \right\}}_{\left(a,b\right)}}d\mu_{0}\left(X\right)=\int_{{\left\{\Psi \right\}}_{\left(a,b\right)}}f_{0}\left(X\right)dX$$

The proof of the transient FR necessitates two lemmas, given in the following.

**Lemma** **A1.**
*Given a time-reversal transformation M satisfying the definition in Equation (6) and the generator of the dynamics of Equation (A1) for a time t, $U_{t}$, the Jacobian of the phase-space coordinate transformation $X^{\prime }=MU_{t}X$ is given by*

(A4) $$\left|J\right|=\left|\frac{dX^{\prime }}{dX}\right|=\exp\left[\int_{0}^{t}ds\Lambda \left(U_{s}X\right)\right]=\exp\left[\Lambda_{0,t}\left(X\right)\right]$$

*where $\Lambda \left(X\right)=\nabla \cdot \dot{X}$ is the phase-space expansion rate and $\Lambda_{0,t}\left(X\right)$ is its time integral over an interval t.*

The well-known proof of the lemma above for M equal to the identity can be found, for example, in Ref. [35]. The transformation M does not substantially modify this proof or explicitly appear in Equation (A4), because it preserves the phase-space volumes, hence $|d{MU}_{t}X/dX|=|d\;U_{t}X/dX|$.

**Lemma** **A2.**
*For any pair of intervals ${\left(\pm A\right)}_{\delta }=\left(\pm A-\delta ,\pm A+\delta \right)\subset R$ with δ>0 and for any τ, the following relation between subsets of M holds*

$${\left\{{\bar{\Psi }}_{0,\tau }\right\}}_{{\left(-A\right)}_{\delta }}=MU_{\tau }{\left\{{\bar{\Psi }}_{0,\tau }\right\}}_{{\left(+A\right)}_{\delta }}$$

*where Ψ is any observable that is odd under the chosen time reversal symmetry M, i.e., Ψ(MX)=−Ψ(X).*

In writing the relation above, we indicate the action of operators on sets of points using the same symbols M and $U_{t}$ adopted for operators acting on phase-space points, meaning that operators acting on a set produce the set of all transformed points under the action of the operators acting on points. For instance, given E⊂M, we take ME as the set of transformed points $\left\{X^{\prime }=MX:X\in E\right\}$.

**Proof.** Let us begin by establishing the action of $MU_{\tau }$ on a subset of phase space. For any interval (a,b)⊂R we may write (see Equation (A3)):

(A5) $$\begin{array}{cc} MU_{\tau }{\left\{\Psi \right\}}_{\left(a,b\right)} & =MU_{\tau }\left\{X\in M:\Psi \left(X\right)\in \left(a,b\right)\right\}=\\ \; & =\{\left(X^{\prime }=MU_{\tau }X\right)\in M:\Psi \left(X\right)\in \left(a,b\right)\}=\\ \; & =\{X^{\prime }\in M:\Psi \left(X={\left(MU_{\tau }\right)}^{-1}X^{\prime }\right)\in \left(a,b\right)\}=\\ \; & =\{X^{\prime }\in M:\Psi \left(U_{-\tau }MX^{\prime }\right)\in \left(a,b\right)\} \end{array}$$

where, in the last equality, we used the properties $U_{\tau }^{-1}=U_{-\tau }$ and $M^{-1}=M$. We will now show that, for any $X\in {\left\{{\bar{\Psi }}_{0,\tau }\right\}}_{{\left(-A\right)}_{\delta }}$, then $MU_{\tau }X\in {\left\{{\bar{\Psi }}_{0,\tau }\right\}}_{{\left(+A\right)}_{\delta }}$. Let us consider the expression for the ${\bar{\Psi }}_{0,\tau }\left(U_{-\tau }MX\right)$ that, based on Equations (A2) and (A5), is the value of the time-averaged observable on a point of the transformed phase-space subset. We have

$${\bar{\Psi }}_{0,\tau }\left(U_{-\tau }MX\right)=\frac{1}{\tau }\int_{0}^{\tau }ds\Psi \left(U_{s}U_{-\tau }MX\right)=\frac{1}{\tau }\int_{0}^{\tau }ds\Psi \left(U_{s-\tau }MX\right)$$

where, in the last equality, the time-composition property of the propagator was employed. Performing the change of variable t=s−τ, the integral becomes

(A6) $${\bar{\Psi }}_{0,\tau }\left(U_{-\tau }MX\right)=\frac{1}{\tau }\int_{-\tau }^{0}dt\Psi \left(U_{t}MX\right)$$

Using the definition of time-reversal transformation, Equation (6), we have that $U_{t}M=MU_{-t}$ and Equation (A6) can be written as

$${\bar{\Psi }}_{0,\tau }\left(U_{-\tau }MX\right)=\frac{1}{\tau }\int_{-\tau }^{0}dt\Psi \left(MU_{-t}X\right)$$

Another change of the integration variable u=−t and the exchange of the integration extrema now yield

(A7) $${\bar{\Psi }}_{0,\tau }\left(U_{-\tau }MX\right)=\frac{1}{\tau }\int_{0}^{\tau }du\Psi \left(MU_{u}X\right)=-\frac{1}{\tau }\int_{0}^{\tau }du\Psi \left(U_{u}X\right)=-{\bar{\Psi }}_{0,\tau }\left(X\right)$$

In going from the second to the third equality, the odd parity of the observable was used, while the last equality recognizes the definition of Equation (A2). From Equation (A7), it immediately follows that if ${\bar{\Psi }}_{0,\tau }\left(U_{-\tau }MX\right)\in \left[-A-\delta ,-A+\delta \right]$ then ${\bar{\Psi }}_{0,\tau }\left(X\right)\in \left[+A-\delta ,+A+\delta \right]$ and vice versa for any phase-space point *X*, which completes the proof. □

The proof shows that the lemma is a direct consequence of time-reversal invariance of the dynamical system under M. We can now derive the following:

**Theorem** **A1** (Transient Ω-FR). *For any pair of intervals ${\left(\pm A\right)}_{\delta }=\left(\pm A-\delta ,\pm A+\delta \right)\subset R$ with δ>0 and for any τ, there exists ε(δ,A,τ) such that*

$$\begin{array}{c} \frac{\mu_{0}\left({\left\{{\bar{\Omega^{\left(0\right)}}}_{0,\tau }\right\}}_{{\left(-A\right)}_{\delta }}\right)}{\mu_{0}\left({\left\{{\bar{\Omega^{\left(0\right)}}}_{0,\tau }\right\}}_{{\left(+A\right)}_{\delta }}\right)}=\exp\left[-\tau [A+\epsilon (\delta ,A,\tau )]\right] \end{array}$$

*with |ϵ(δ,A,τ)|≤δ.*

**Proof.** We shall consider first a generic observable and then specialize to the case of the dissipation function. Let us then consider an observable Ψ which is odd under the time-reversal transformation M, i.e., Ψ(MX)=−Ψ(X). The ratio between the probabilities to find the value of its time average for a time τ of the observable in a neighborhood of size δ of certain values +A and −A can be written as

(A8) $$\begin{array}{c} \frac{\mu_{0}\left({\left\{{\bar{\Psi }}_{0,\tau }\right\}}_{{\left(-A\right)}_{\delta }}\right)}{\mu_{0}\left({\left\{{\bar{\Psi }}_{0,\tau }\right\}}_{{\left(+A\right)}_{\delta }}\right)}=\frac{\int_{{\left\{{\bar{\Psi }}_{0,\tau }\right\}}_{{\left(-A\right)}_{\delta }}}f_{0}\left(X\right)dX}{\int_{{\left\{{\bar{\Psi }}_{0,\tau }\right\}}_{{\left(+A\right)}_{\delta }}}f_{0}\left(X\right)dX} \end{array}$$

Using Lemma A2

(A9) $$\begin{array}{c} {\left\{{\bar{\Psi }}_{0,\tau }\right\}}_{{\left(-A\right)}_{\delta }}=MU_{\tau }{\left\{{\bar{\Psi }}_{0,\tau }\right\}}_{{\left(+A\right)}_{\delta }} \end{array}$$

the domain of integration in the numerator of the RHS of Equation (A8) can be written as

(A10) $$\begin{array}{c} \int_{{\left\{{\bar{\Psi }}_{0,\tau }\right\}}_{{\left(-A\right)}_{\delta }}}f_{0}\left(X\right)dX=\int_{MU_{\tau }{\left\{{\bar{\Psi }}_{0,\tau }\right\}}_{{\left(+A\right)}_{\delta }}}f_{0}\left(X\right)dX \end{array}$$

following which the integral at the numerator can be expressed as

(A11) $$\begin{array}{c} \frac{\mu_{0}\left({\left\{{\bar{\Psi }}_{0,\tau }\right\}}_{{\left(-A\right)}_{\delta }}\right)}{\mu_{0}\left({\left\{{\bar{\Psi }}_{0,\tau }\right\}}_{{\left(+A\right)}_{\delta }}\right)}=\frac{\int_{{\left\{{\bar{\Psi }}_{0,\tau }\right\}}_{{\left(+A\right)}_{\delta }}}f_{0}\left(MU_{\tau }X\right)d\left(MU_{\tau }X\right)}{\int_{{\left\{{\bar{\Psi }}_{0,\tau }\right\}}_{{\left(+A\right)}_{\delta }}}f_{0}\left(X\right)dX} \end{array}$$

It is now possible to recognize in the integral in the numerator of the RHS of Equation (A11) a change of variables of the kind $X^{\prime }=MU_{\tau }X$. We can then apply Lemma A1 so that Equation (A8) takes the form

(A12) $$\frac{\mu_{0}\left({\left\{{\bar{\Psi }}_{0,\tau }\right\}}_{{\left(-A\right)}_{\delta }}\right)}{\mu_{0}\left({\left\{{\bar{\Psi }}_{0,\tau }\right\}}_{{\left(+A\right)}_{\delta }}\right)}=\frac{\int_{{\left\{{\bar{\Psi }}_{0,\tau }\right\}}_{{\left(+A\right)}_{\delta }}}f_{0}\left(MU_{\tau }X\right)\exp\left[\Lambda_{0,\tau }\left(X\right)\right]dX}{\int_{{\left\{{\bar{\Psi }}_{0,\tau }\right\}}_{{\left(+A\right)}_{\delta }}}f_{0}\left(X\right)dX}$$

If the equilibrium density $f_{0}$ is even under the time-reversal transformation M, i.e., $f_{0}\left(MX\right)=f_{0}\left(X\right)$, the ratio of the probabilities is then given by

(A13) $$\frac{\mu_{0}\left({\left\{{\bar{\Psi }}_{0,\tau }\right\}}_{{\left(-A\right)}_{\delta }}\right)}{\mu_{0}\left({\left\{{\bar{\Psi }}_{0,\tau }\right\}}_{{\left(+A\right)}_{\delta }}\right)}=\frac{\int_{{\left\{{\bar{\Psi }}_{0,\tau }\right\}}_{{\left(+A\right)}_{\delta }}}f_{0}\left(U_{\tau }X\right)\exp\left[\Lambda_{0,\tau }\left(X\right)\right]dX}{\int_{{\left\{{\bar{\Psi }}_{0,\tau }\right\}}_{{\left(+A\right)}_{\delta }}}f_{0}\left(X\right)dX}$$

To proceed, we now consider the time integral of $\Omega^{\left(0\right)}$ from Equation (8) in the interval 0,τ

(A14) $$\Omega_{0,\tau }^{\left(0\right)}\left(X\right)=\ln\left[\frac{f_{0}\left(X\right)}{f_{0}\left(U_{\tau }X\right)}\right]-\Lambda_{0,\tau }\left(X\right)$$

The result above is obtained by observing that the first term on the RHS of Equation (8) is the total time derivative of the logarithm of $f_{0}$ and that $\Lambda_{0,\tau }\left(X\right)$ denotes the time integral of the second term. Using Equation (A14) and multiplying and dividing the integrand in the numerator by $f_{0}\left(X\right)$, Equation (A8) can be recast in the form

(A15) $$\frac{\mu_{0}\left({\left\{{\bar{\Psi }}_{0,\tau }\right\}}_{{\left(-A\right)}_{\delta }}\right)}{\mu_{0}\left({\left\{{\bar{\Psi }}_{0,\tau }\right\}}_{{\left(+A\right)}_{\delta }}\right)}=\frac{\int_{{\left\{{\bar{\Psi }}_{0,\tau }\right\}}_{{\left(+A\right)}_{\delta }}}f_{0}\left(X\right)\exp\left[-\Omega_{0,\tau }^{\left(0\right)}\right]dX}{\int_{{\left\{{\bar{\Psi }}_{0,\tau }\right\}}_{{\left(+A\right)}_{\delta }}}f_{0}\left(X\right)dX}≐{\langle \exp\left[-\Omega_{0,\tau }^{\left(0\right)}\right]\rangle }_{{\left\{{\bar{\Psi }}_{0,\tau }\right\}}_{{\left(+A\right)}_{\delta }}}^{\left(0\right)}$$

where the last equality defines the conditional phase-space average of $\exp\left[-\Omega_{0,\tau }^{\left(0\right)}\right]$ with respect to the measure $\mu_{0}$, over the set of initial conditions *X* such that ${\bar{\Psi }}_{0,\tau }\in {\left(+A\right)}_{\delta }$. We now move to the case $\Psi =\Omega^{\left(0\right)}$ to obtain

(A16) $$\frac{\mu_{0}\left({\left\{{\bar{\Omega^{\left(0\right)}}}_{0,\tau }\right\}}_{{\left(-A\right)}_{\delta }}\right)}{\mu_{0}\left({\left\{{\bar{\Omega^{\left(0\right)}}}_{0,\tau }\right\}}_{{\left(+A\right)}_{\delta }}\right)}={\langle \exp\left[-\Omega_{0,\tau }^{\left(0\right)}\right]\rangle }_{{\left\{{\bar{\Omega^{\left(0\right)}}}_{0,\tau }\right\}}_{{\left(+A\right)}_{\delta }}}^{\left(0\right)}$$

One final observation takes us to the usual form of the transient FR given in Section 2: The average above is conditioned on ${\bar{\Omega^{\left(0\right)}}}_{0,\tau }$ taking values in ${\left(+A\right)}_{\delta }=\left(+A-\delta ,+A+\delta \right)$. This implies that ${\Omega^{\left(0\right)}}_{0,\tau }$ at the exponent of the right hand side can be approximated as Aτ plus a correction term ϵ(δ,A,τ) not larger than δ. Thus,

(A17) $$\frac{\mu_{0}\left({\left\{{\bar{\Omega^{\left(0\right)}}}_{0,\tau }\right\}}_{{\left(-A\right)}_{\delta }}\right)}{\mu_{0}\left({\left\{{\bar{\Omega^{\left(0\right)}}}_{0,\tau }\right\}}_{{\left(+A\right)}_{\delta }}\right)}={\langle \exp\left[-\Omega_{0,\tau }^{\left(0\right)}\right]\rangle }_{{\left\{{\bar{\Omega^{\left(0\right)}}}_{0,\tau }\right\}}_{{\left(+A\right)}_{\delta }}}^{\left(0\right)}\;\;\;\;\;\;\;\;\;=\exp\left[-[A+\epsilon (\delta ,A,\tau )]\tau \right]$$

□

Note that the only requirements for the proof above are invariance of the dynamics and of $f_{0}$ under M, and that the dissipation is odd under the same time-reversal. The specific form of the time-reversal operator M is irrelevant, as long as Equation (6) is satisfied.

## Appendix B. Derivation of the Dissipation Function for the Generalized Nosè–Hoover Thermostatted System

The dissipation function $\Omega^{\left(0\right)}\left(X\right)$ for the dynamical system (3) is obtained from the definition (8)

$$\Omega^{\left(0\right)}\left(X\right)=-\dot{X}\cdot \nabla_{X}\ln f_{0}-\nabla_{X}\cdot \dot{X}$$

substituting $\dot{X}$ from Equations (3) and the expression for $f_{0}$ from Equation (4). Let us consider first the term $\nabla_{X}\cdot \dot{X}$. Noting that the variable *s* does not enter the dynamics we have $\nabla_{X}\cdot \dot{X}=\nabla_{\Gamma }\cdot \dot{\Gamma }+\nabla_{\xi }\dot{\xi }=\nabla_{\Gamma }\cdot \dot{\Gamma }$ where the last equality holds because the variable ξ is not involved in the evolution equation for $\dot{\xi }$. The evolution equations of the physical variables in the dynamical system (3) can be written, in vector notation, as

(A18) $$\begin{array}{cc} {\dot{r}}_{i} & =\nabla_{p_{i}}H\\ {\dot{p}}_{i} & =-\nabla_{r_{i}}H-\xi \left(p_{i}-q_{i}A\left(r_{i}\right)\right) \end{array}$$

where the explicit form of *H* is given in Equation (2). Using this writing it is easy to show that

(A19) $$\begin{array}{cc} \nabla_{X}\cdot \dot{X} & =\nabla_{\Gamma }\cdot \dot{\Gamma }=\sum_{i}^{N}\left[\nabla_{r_{i}}\cdot {\dot{r}}_{i}+\nabla_{p_{i}}\cdot {\dot{p}}_{i}\right]\\ \; & =\sum_{i}^{N}\left[\nabla_{r_{i}}\cdot \nabla_{p_{i}}H-\nabla_{p_{i}}\cdot \nabla_{r_{i}}H-\xi \nabla_{p_{i}}\left(p_{i}-q_{i}A\left(r_{i}\right)\right)\right]=-G\xi \end{array}$$

where G=3N. We now move to the calculation of the first term in the definition of the dissipation function:

(A20) $$\dot{X}\cdot \nabla_{X}\ln f_{0}\left(X\right)=\dot{X}\cdot \frac{1}{f_{0}}\nabla_{X}f_{0}\left(X\right)$$

We have

(A21) $$\frac{1}{f_{0}}\nabla_{X}f_{0}\left(X\right)=\frac{1}{f_{0}}\left(\begin{array}{c} \nabla_{\Gamma }\\ \nabla_{\xi } \end{array}\right)Z^{-1}\exp\left[-\beta H_{0}\left(\Gamma \right)\right]\exp\left[-\frac{G\tau_{\mathrm{NH}}^{2}\xi^{2}}{2}\right]=\left(\begin{array}{c} -\beta \nabla_{\Gamma }H_{0}\left(\Gamma \right)\\ -G\tau_{\mathrm{NH}}^{2}\xi \end{array}\right)$$

so that

(A22) $$\dot{X}\cdot \frac{1}{f_{0}}\nabla_{X}f_{0}\left(X\right)=-\beta \dot{\Gamma }\cdot \nabla_{\Gamma }H_{0}\left(\Gamma \right)-G\tau_{\mathrm{NH}}^{2}\dot{\xi }\xi$$

The last term in the equation above is directly computed using the expression of $\dot{\xi }$ given in Equation (3) to obtain

(A23) $$-G\tau_{\mathrm{NH}}^{2}\xi \dot{\xi }=-G\xi \delta K\left(\Gamma \right)$$

where $\delta K(\Gamma )=\left[\frac{K\left(\Gamma \right)-K^{*}}{K^{*}}\right]$, with K(Γ) the kinetic energy of the system and $K^{*}=G/2\beta$ (see Section 2). To compute the first term of the RHS of Equation (A22) it is convenient to write the evolution equations for the physical variables (see system (3)) in the form

(A24) $$\begin{array}{cc} {\dot{r}}_{i} & =\nabla_{p_{i}}H_{0}\\ {\dot{p}}_{i} & =-\nabla_{r_{i}}H_{0}+q_{i}E-\xi \left(p_{i}-q_{i}A\left(r_{i}\right)\right) \end{array}$$

with $H_{0}$ defined in Equation (2). Using the equations above, we have

(A25) $$\begin{array}{cc} -\beta \dot{\Gamma }\cdot \nabla_{\Gamma }H_{0}\left(\Gamma \right) & =-\beta \sum_{i}^{N}\left[{\dot{r}}_{i}\cdot \nabla_{r_{i}}H_{0}+{\dot{p}}_{i}\cdot \nabla_{p_{i}}H_{0}\right]\\ \; & =-\beta \sum_{i}^{N}\left[q_{i}E\cdot \nabla_{p_{i}}H_{0}-\xi \left(p_{i}-q_{i}A\left(r_{i}\right)\right)\cdot \nabla_{p_{i}}H_{0}\right] \end{array}$$

From the first of Equation (A24) we have $\nabla_{p_{i}}H_{0}={\dot{r}}_{i}$, so that substituting in the first term of the RHS of Equation (A25) we get

(A26) $$-\beta \sum_{i}^{N}q_{i}E\cdot \nabla_{p_{i}}H_{0}=-\beta \sum_{i}^{N}q_{i}E\cdot {\dot{r}}_{i}=-\beta VJ\left(\Gamma \right)\cdot E$$

where the microscopic estimator of the electric current $J\left(\Gamma \right)=\sum_{i}^{N}q_{i}{\dot{r}}_{i}/V$ has been introduced, together with the box volume V, as in Section 2. The second term in the RHS of Equation (A25) is easily computed substituting $\nabla_{p_{i}}H_{0}=m_{i}^{-1}\left(p_{i}-q_{i}A\left(r_{i}\right)\right)$ in its expression

(A27) $$\xi \beta \sum_{i}^{N}\left(p_{i}-q_{i}A\left(r_{i}\right)\right)\cdot \nabla_{p_{i}}H_{0}=\xi \beta \sum_{i}^{N}\frac{{\left(p_{i}-q_{i}A\left(r_{i}\right)\right)}^{2}}{m_{i}}=\xi \beta 2K\left(\Gamma \right)$$

where, in the last equality, we used the definition of the kinetic energy of the system, K(Γ). Combining Equations (A26) and (A27), we then get $-\beta \dot{\Gamma }\cdot \nabla_{\Gamma }H_{0}\left(\Gamma \right)=-\beta VJ\left(\Gamma \right)\cdot E+\xi \beta 2K\left(\Gamma \right)$. Using this result together with Equation (A23), Equation (A22) can be rewritten as

(A28) $$\dot{X}\cdot \frac{1}{f_{0}}\nabla_{X}f_{0}\left(X\right)=-\beta VJ\left(\Gamma \right)\cdot E+\xi \beta 2K\left(\Gamma \right)-G\xi \delta K\left(\Gamma \right)$$

Substituting Equations (A28) and (A19) in the definition of the dissipation function we get

(A29) $$\Omega^{\left(0\right)}\left(X\right)=-\dot{X}\cdot \nabla_{X}\ln f_{0}-\nabla_{X}\cdot \dot{X}=\beta VJ\left(\Gamma \right)\cdot E-\xi \beta 2K\left(\Gamma \right)+G\xi \delta K\left(\Gamma \right)+G\xi$$

Using the definitions of δK(Γ) and of $K^{*}$ and after trivial simplifications, we finally obtain

(A30) $$\begin{array}{cc} \Omega^{\left(0\right)}\left(X\right) & =\beta VJ(\Gamma )\cdot E \end{array}$$

which is the result given in Section 2.

We conclude this Appendix showing that, as indicated in Section 2, the microscopic kinetic energy provides an estimator of the temperature also in the presence of an external magnetic field in the equilibrium ensemble characterized by the canonical equilibrium density $f_{0}$. To proceed, let us consider

(A31) $$\langle K\rangle =\frac{1}{Z}\int d\Gamma \left(\sum_{i}^{N}\frac{{\left(p_{i}-q_{i}A\left(r\right)\right)}^{2}}{2m_{i}}\right)\exp\left[-\beta \left(\sum_{i}^{N}\frac{{\left(p_{i}-q_{i}A\left(r\right)\right)}^{2}}{2m_{i}}+V\left(r\right)\right)\right]$$

with $Z=\int d\Gamma \exp\left[-\beta \left(\sum_{i}^{N}\frac{{\left(p_{i}-q_{i}A\left(r\right)\right)}^{2}}{2m_{i}}+V\left(r\right)\right)\right]$, the partition function. In the absence of the magnetic field, i.e., for A(r)=0, the expression above reduces to the standard kinetic energy estimator of the temperature. When A(r)≠0, the estimator can be identified by performing the change of variables $p_{i}^{\prime }=p_{i}-q_{i}A\left(r\right)$, $r_{i}^{\prime }=r$. (The Jacobian for this change of variables is one.) In the new variables, the integrals over $r_{i}^{\prime }$ and $p_{i}^{\prime }$ are independent, and the integral over positions can be trivially simplified with the same one in the denominator. Indicating with $Z_{p^{\prime }}$ the remaining normalization, we then have

(A32) $$\langle K\rangle =\frac{1}{Z_{p^{\prime }}}\int d^{3}p_{1}^{\prime }\cdots d^{3}p_{N}^{\prime }\left(\sum_{i}^{N}\frac{{\left(p_{i}^{\prime }\right)}^{2}}{2m_{i}}\right)\exp\left[-\beta \sum_{i}^{N}\frac{{\left(p_{i}^{\prime }\right)}^{2}}{2m_{i}}\right]=\frac{G}{2\beta }$$

in complete analogy with the standard case.
